# Climate-Driven Variation in the Intensity of a Host-Symbiont Animal Interaction along a Broad Elevation Gradient

**DOI:** 10.1371/journal.pone.0101942

**Published:** 2014-07-15

**Authors:** Leandro Meléndez, Paola Laiolo, Sergey Mironov, Mónica García, Oscar Magaña, Roger Jovani

**Affiliations:** 1 Research Unit of Biodiversity (UO, CSIC, PA), Oviedo University, Campus de Mieres, Mieres, Spain; 2 Department of Evolutionary Ecology, Estación Biológica de Doñana (CSIC), Avda, Americo Vespucio s/n, Sevilla, Spain; 3 Zoological Institute, Russian Academy of Sciences, Universitetskaya Embankment 1, Saint Petersburg, Russia; University of Northampton, United Kingdom

## Abstract

Gradients of environmental stress may affect biotic interactions in unpredictable ways responding to climate variation, depending on the abiotic stress tolerance of interacting partners. Here, we study the effect of local climate on the intensity of feather mites in six mountain passerines along a 1400 m elevational gradient characterized by shifting temperature and rainfall. Although obligatory symbionts of warm-blooded organisms are assumed to live in mild and homeothermic environments, those inhabiting external, non-blood-irrigated body portions of the host organism, such as feather mites, are expected to endure exposure to the direct influence of a fluctuating climate. As expected, feather mite intensity declined with elevation in all bird species, a pattern that was also found in cold-adapted passerines that have typical alpine habits. The elevation cline was mainly explained by a positive effect of the average temperature upon mite intensity in five of the six species studied. Precipitation explained less variance in mite intensity than average temperature, and showed a negative correlation in half of the studied species. We found no climate-driven migration of mites along the wings of birds, no replacement of mite species along elevation gradients and no association with available food resources for mites (estimated by the size of the uropygial gland). This study suggests that ectosymbionts of warm-blooded animals may be highly sensitive to climatic variation and become less abundant under stressful environmental conditions, providing empirical evidence of the decline of specialized biotic interactions among animal species at high elevations.

## Introduction

Climate is the most general abiotic factor that governs organism ecology, shaping from life histories to species distribution patterns [1], [2], [3]. Its influence may be direct, on individual metabolism and development, or indirect, when it alters habitats or the way species interact [4], [5], [6]. Previous studies have suggested that the frequency of biotic interactions may decline when environmental conditions become harsher (“Stress Gradient Hypothesis” [7], [8], [9]). For instance, it has been found that the number and/or prevalence of parasites could be higher in warm compared to cold environments [10], and similar patterns occur in other types of biotic interactions [11]. These gradients have been most often described in facultative or non-specialized interactions, but less is known about the responses to climate gradients in species involved in obligatory specialized interactions, i.e. interactions in which at least one partner fully depends on a specific counterpart [12]. These interactions are assumed to respond to abiotic changes more slowly than unspecialized ones, since the obligate partner first depend upon the other for survival, maintenance and reproduction, and may only be indirectly influenced by environmental influences outside of its partner [13]. However, when partners respond differently to environmental stressors, the outcome of the interaction can suffer immediate and serious consequences, with a disruption of temporal or geographical synchronization [14].

Changes in symbiont species abundance, composition, or performance along gradients of temperature and rainfall have been documented in plant interactions with bacteria, fungi, and animals [15], [16], [17]. Symbiotic interactions between animal species have received less attention and knowledge is largely biased toward parasitoid systems, where it has been shown that extreme temperatures reduce endosymbiont populations, and can eventually eliminate those populations that are most susceptible to climatic shifts [18]. In this study we address the outcome of a symbiotic association along an elevation gradient of precipitation and temperature, analyzing the variation in the intensity of feather mites on open-habitat birds. Mountains show dramatic climatic shifts and are often used as “field laboratories” to analyze the effect of abiotic factors on species' ecology, since certain climatic features (e.g. temperature) show straightforward and predictable patterns of variation and others (e.g. humidity and rainfalls) may be adequately predicted at the local scale when accounting for latitude, distance from the sea and local topography [19].

Plumicolous feather mites (Astigmata: Analgoidea, Pterolichoidea) are obligate symbiotic arthropods living permanently on the feathers of live birds, and are their most common and numerous invertebrates inhabiting on these hosts [20], [21], [22]. Their ectothermic condition predisposes them to be highly dependent on external conditions, and temperature and humidity have been repeatedly found to be important determinants of their physiology [23], [24], [25], [26], [22]. Birds have an average body temperature of 42–45°C and Dubinin [23] proposed that feather mites show a peak of activity at 40°C, becoming inactive below 15°C. Dubinin [23] also suggested that optimum air humidity for feather mites is around 10–20%. However, this is at odds with other evidence. Bird feathers do not trap humid air from the bird's body water vapor, being highly correlated with the environment humidity [27]. The diet of feather mites seems to provide an unreliable water supply, and thus they take up water vapor from the surrounding air into the hemolymph through different mechanisms. In *Proctophyllodes troncatus* living in house sparrows (*Passer domesticus*), it has been experimentally shown that they can do this when surrounding air exceeds a critical humidity threshold around 55–60% [25]. While there is no similar information for other feather mite species, this indicates that the higher air humidity would be positive for feather mites. This would be in agreement with what has been found in feather lice, which also have a dry diet, live in the same environment, and have the same capacity for uptake of water vapor from the air [27].

Mite population dynamics is crucially affected by the interaction with the host. Some intraspecific and comparative studies have shown that mite intensity is higher in birds with larger uropygial glands, owing to the fact that larger-sized glands seem to produce higher quantities of lipids. These lipids are spread onto plumage by birds during the preening activity and are a food resource for mites [28], [29], [30], [31]. Mite intensity can also vary in response to bird body size, body condition, age and sex [32], [33], [34], [35], [36]. Although some authors suggested that the relationship between feather mites and birds might be parasitic [37], [38], [39], [40], the nature of this interaction is still poorly understood. Several studies suggested, for instance, that mites may benefit birds by removing old waxes and preying upon keratinophagous micro-organisms [32], [33], [35], [36], [41].

In the present study, we analyzed changes in the intensity of feather mites in a bird mountain community composed by six common passerines of open habitats along an altitudinal gradient of almost 1,400 m. We tested for the influence of local average temperature and precipitation on mite intensity along an elevation gradient, testing whether at the harsh extreme of the stress gradient (lower temperatures or humidity) biotic interactions become less intense. Additionally, by focusing on the most abundant species (the water pipit, *Anthus spinoletta*) we analyzed the role of three possible confounding factors that could bias mite intensity estimates along elevation irrespective of climate. The first of these factors was feather mite movements within the wing of birds to reach less exposed sites at high elevations, since mites can retreat from cold environmental conditions by migrating towards inner wing flight and cover feathers [23], [26]. If this is the case, feather mites would be more difficult to find on birds from high elevations because of mite migration towards more protected areas. To account for this, we analyzed the distribution of feather mites along the wing feathers with respect to elevation at varying instant air temperature. A shift of mite distribution within the wing flight feathers would be indicative that temperature could alter mite distribution rather than actually affect mite population sizes. Second, intensity patterns along elevation could be affected by the replacement of mite species with distinct climate preferences at different altitudes, as commonly observed in plant symbionts along climatic clines [42]. To explore this possibility we studied variation in feather mite community composition along the whole altitudinal gradient. We also accounted for uropygial gland size, since this could affect resource availability for mites [29].

## Materials and Methods

### Ethics statement

The study was conducted in compliance with the current laws of the Spanish Government and scientific permits issued by the Picos de Europa National Park (permit numbers: CO/09/0559/2009 CO/09/033/2011; CO/08/058/2012; CO/09/016/2013), Gobierno del Principado de Asturias (2012/006078), Gobierno de Cantabria (SEP/142.2/09) and Junta de Castilla y Leon (EP/CYL/239/2009). Bird ringing was done by LM under the Spanish Ministry of Agriculture, Food and Environment ringing licence number 530406.

### Study area and host bird species

**Figure 1:**
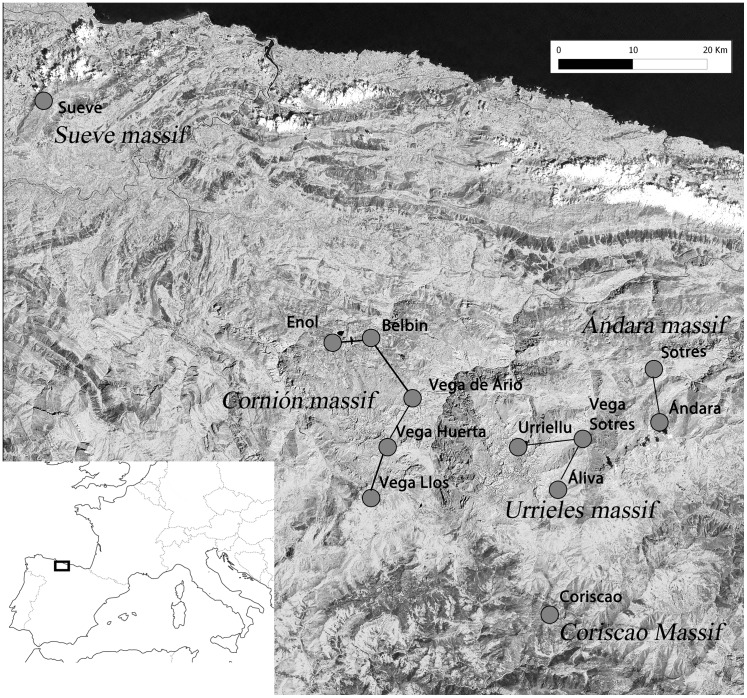
The study area. Map of the study area, depicting the five massifs and the 12 capture localities (grey dots).

**Figure 2:**
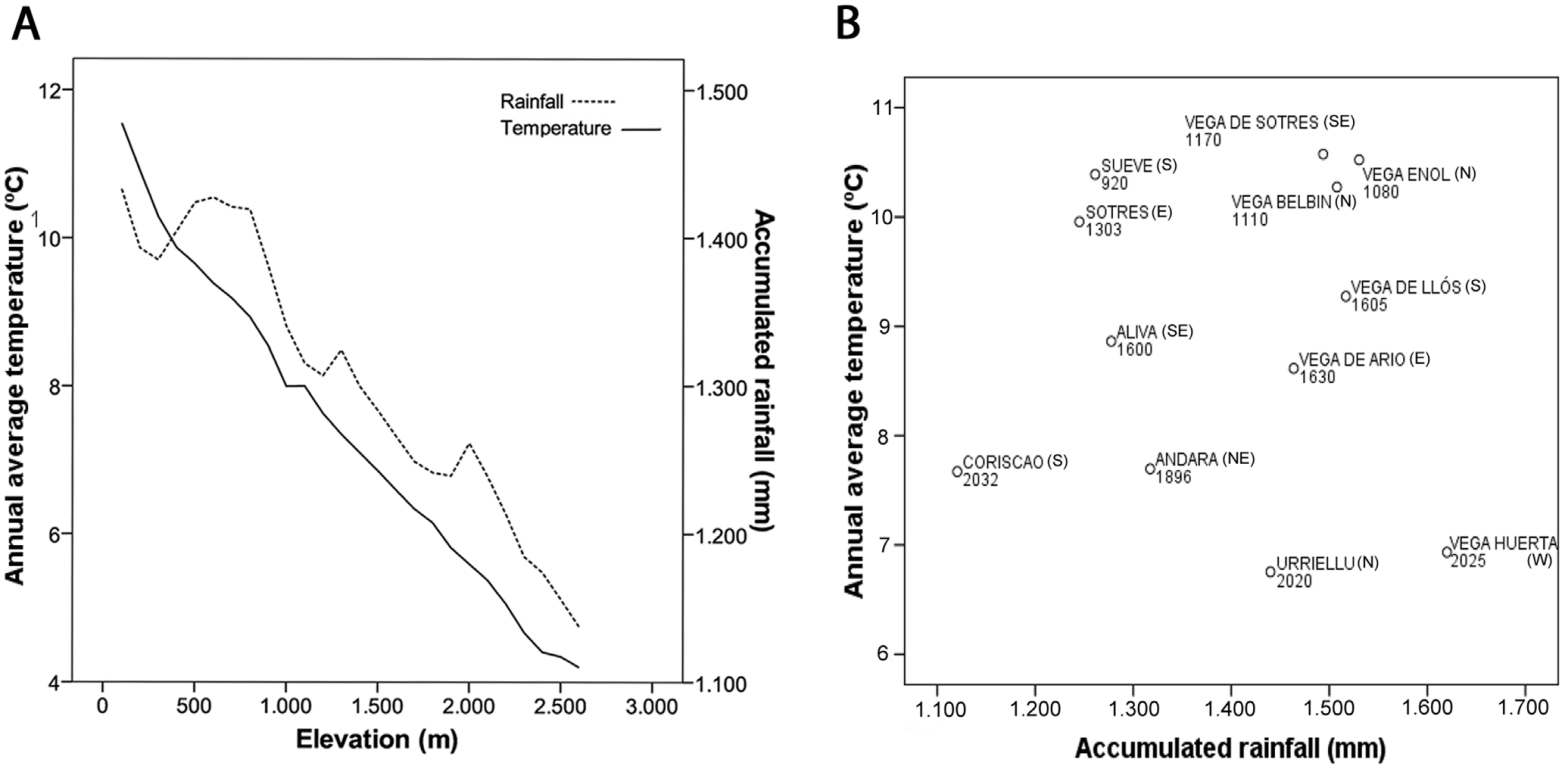
Climate of the Study area and localities. (A) Graphical representation of the average annual temperature (°C; solid line) and accumulated rainfall (millimeters/year; dotted line) along the elevational gradient in our study area. (B) Annual average temperature and annual accumulated rainfall of each sampling locality, and the associated elevation (m.a.s.l.). The slope exposition is also shown (N  =  North, S  =  South, E  =  East, W  =  West).

The study was carried out in the Cantabrian Mountains, northern Spain, from May to July 2009–2013. Our study area was located in the highest portions of this mountain chain, in four massifs within the Picos de Europa National Park (43°07′- 43°16′N, 5°01′- 4°39′W, highest peak at 2648 m a.s.l.), which cover 16.925 ha (Fig. 1). This area is characterized by a steep elevational gradient, with a vertical rise of 2400 m in very short linear distances. The local climate is wet because of the influence of the Atlantic, particularly on the north-western slopes. Precipitation values range from 1100 to 1800 mm per year, and the average temperature from 4°C to 11°C per month (Fig. 2).

We studied six common passerine species widely distributed along the elevational gradient: an exclusively alpine species (the alpine accentor, *Prunella collaris*), two high-elevation species also breeding in montane grasslands (the water pipit, and the northern wheatear, *Oenanthe oenanthe*) and three generalist species from open habitats (the linnet, *Carduelis cannabina,* the black redstart, *Phoenicurus ochruros,* and the dunnock, *Prunella modularis*). The studied bird species inhabit open habitats, mainly pastures and alpine prairies with interspersed rocky outcrops. With the exception of the northern wheatear, which is a long distance migrant, the other five species are resident species, performing only short winter elevational displacements. Birds were sampled by using mist nets and bird mesh traps in 12 localities distributed homogeneously along an elevational range from 850 m to 2200 m. The sample localities were located on different slopes of the four massifs to account for the greatest variability in climatic conditions (Fig. 2b). Elevation and UTM position of captured birds were measured with a GPS (Garmin eTrex Summit HC). All birds were banded and biometric measurements were taken according to Svensson [43]. Age was determined from plumage characteristics and sex by the inspection of the cloacal protuberance (in males) and the brood patch (in females) [43].

### Feather mite counts and sampling

In the present study, we investigated Proctophyllodidae feather mites specialized to live on large and firm vanes of the flight feathers of birds. These mites were visually counted in all primary, secondary and tertiary feathers by extending and exposing the right wing of birds to daylight [32], [35]. Mite intensity was estimated as the number of mites per wing in infected birds (thus always greater than zero). We did not count mites in moulting wing feathers to exclude known disturbance of moulting upon mite distribution [23], [44], [45]. All mite sampling and counts were done by the same observer (LM), from May to July 2009–2013. Each breeding season, sampling started at lower elevations and ended at the tops, thus sampling birds in same breeding stage along this broad elevational gradient [46]. When counting mites we never observed any movement of mites within or between feathers. Thus, although we cannot discard some short distance displacement of mites during capture and handling, we can reasonably argue that this does not affect abundance differences among localities since all caught birds were handled following the same protocol at all study localities (involving counting mites, taking morphometric measurements and ringing the birds, for a total of 7–10 minutes per bird).

In the water pipit, mite samples were retrieved with a cotton swab soaked in 98% ethanol by swabbing all primary, secondary and tertiary feathers that were hosting mites. Mites were stored in 98% ethanol until inspection. The distribution of feather mites within the wing of water pipits was recorded by counting the number of mites on each of the 19 flight feathers of the wing (10 primaries, 6 secondary and 3 tertiary feathers). Only water pipit adults with more than ten feather mites on the wing were selected for the analysis of feather mite distribution. The uropygial gland size of the water pipit was estimated by soaking the surrounding feathers with moist cotton and measuring its maximum length and width with a digital calliper to the nearest 0.01 mm according to Galván and Sanz [29].

### Visual identification of mites

For specific identification of mites from water pipits, mites stored in 98% ethanol were mounted on microslides in Faure medium according to the standard technique used for astigmatan and other small-sized mites [47]. A few male individuals from each sample were mounted and then identified under a DMLS Leica microscope. Generic identification was based on the monograph by Gaud and Atyeo [48], and species identification was made using the key to the species of the genus *Proctophyllodes* [49] and species descriptions by Černý [50].

### Climatic variables

The instant air temperature was measured at ringing by means of a digital thermometer to the nearest 0.1°C that was maintained in a shaded area and 50 cm far off the ground. Moreover, we considered maximum and minimum annual temperature, and annual accumulated rainfall; for the breeding period (March to August, i.e. from territory occupancy to the last breeding attempts) we calculated average temperature and accumulated rainfall. Climatic variables were obtained from layers of the Climate Atlas of the Iberia Peninsula (resolution 200 m [51]), extracting values for the coordinates of each mist net and mesh trap where each bird was captured. The climatic Atlas results from a model of 15 years of meteorological data from the local Meteorological National Institute stations. Since we found a high correlation between average monthly temperatures and between monthly precipitations (temperature variables for six months: 0.74<r<0.99, all *P*<0.01; precipitation variables for six months: 0.59<r<0.96, all *P*<0.01) we considered the average temperature from March to August as an indicator of temperature during territory occupancy, and the accumulated rainfall in the same period as an indicator of rainfall during breeding.

### Data analysis

Two sets of six generalized linear mixed models (GLMMs), i.e. one for each species, were constructed to test how mite intensity correlates with either elevation or climate. The sex and age of birds were grouped in three categories (adult males, adult females and juveniles) and included as a fixed factor in both elevation and climate models (the juvenile class was not included in dunnock models due to insufficient sample size). Massif identity was entered as a random factor to account for data pseudoreplication and unequal sampling among massifs. Wing length (or tarsus length in the case of the alpine accentor, due to strong abrasion of wing feathers at the time of the study) was also introduced as a covariate in order to control for body size [36]. The reason for controlling for body size is that a positive correlation between mite intensity and body size could arise because larger host species and individuals within species have larger surfaces of flight feathers and thus can harbor more mites [34], [35]. Previous studies suggested that mite abundance could change with individual body condition [36]. However, we discarded this variable as a covariate as we only found change in bird individual condition across elevations in two of the six bird species studied (as measured by the residuals of bird weight on wing or tarsus length) (Table S1). Only individuals with one or more feather mites were included in all models (i.e., we analyzed the intensity of mites). This is because the absence of feather mites on a bird could indicate either the influence of climate factors (or other factors) or that the individual never had feather mites (e.g., if parents had no feather mites and thus did not transmit them to the offspring). A Poisson distribution of errors was used for both sets of mite intensity models. We also estimated the relative importance of each climatic variable of the climatic model by calculating the percentage of variance explained by each variable (running separate models for average temperature and precipitation) comparing them with the variance explained by the full model with both climatic variables. Percentage of explained variance was calculated from the deviance (*D*) estimates, as follows: Variance explained for the variable x (%) = [(*D* null model – *D* model x)/*D* null model)] ×100.

To test for a possible change on the spatial distribution of mites, we tested for differences in the percentages of mites occupying primaries (distal wing feathers) and tertiaries (proximal wing feathers) along the elevation gradient and at different instant air temperatures with a non-parametric Friedman's test for repeated measures. Since only the percentage of mites in primaries, and not tertiaries, followed a Gaussian distribution (Lillie test: *D* = 0.076, *P* = 0.18) Spearman's correlation tests were used to analyse variation with elevation. The relationships between uropygial gland size and elevation, and between feather mite intensity and gland size, were addressed by means of GLMMs, in which wing length and sex-age class were entered as covariates and massif identity as a random factor. A Gaussian distribution of errors was used in both models, since the dependent variables were normally distributed (Lillie test, gland size: *P* = 0.501, mite intensity: *P* = 0.130).

All the analyses were performed with the software R, version 2.15 [52]; the package *lme4* was used for GLMMs. We performed SS type III models, presenting partial effects of the variables by controlling for the effect of one variable over all others [53].

## Results

**Figure 3:**
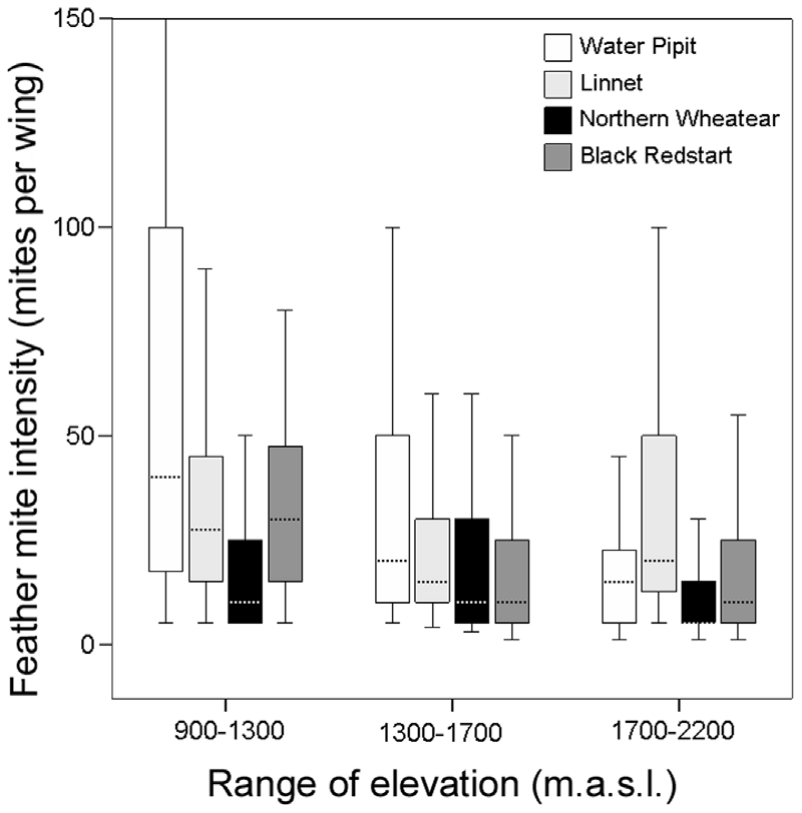
Feather mite intensity along elevation. Box-plot of feather mite intensity along the elevation gradient. Only the four best sampled species are shown.

Feather mite intensity was highly variable between and within bird species (Table 1). We found a consistent decline on the intensity of feather mites at higher elevations in all bird species (Table 2, Fig. 3). The intensity of mites was higher in adults than in juveniles in all species, and higher in adult females than in males in all species with the exception of the linnet, where the opposite was found (Table 2). Wing length was positively associated with mite intensity in all bird species (Table 2).

**Table 1:** Exploratory analysis of feather mite intensity in the six species studied.

	Range	Mean	Median	SD	Prevalence	n	Elevation
Water pipit	1–375	49.25	20	63.46	92.90	423	900–2175
Northern wheatear	1–300	21.77	5	36.76	78.90	194	894–2175
Black redstart	1–120	27.22	10	30.60	76.00	307	905–2175
Linnet	4–400	38.98	20	45.78	93.02	219	900–2175
Dunnock	1–160	15.56	5	21.57	87.20	73	902–2014
Alpine accentor	1–220	37.40	30	66.40	97.64	85	1616–2200

The table present the results of the range of feather mite intensity, mean, median intensity, standard deviation (SD) and prevalence (percentage of individuals with at least one feather mite counted), for the six bird species studied. The number of birds sampled and the elevational range (m.a.s.l.) of captures are also shown.

**Table 2:** Results of generalized linear mixed models testing for the relationship between mite intensity and elevation.

		Estimate	SE	*Z*	*P*
**water pipit**	Elevation	−0.002	3.74×10^−5^	−28.528	<0.001
n = 387	Wing length	0.036	0.003	11.353	<0.001
Age-sex class:	Adult Male	0.000	-	-	-
	Adult Female	0.161	0.030	5.285	<0.001
	Juvenile	−0.525	0.0241	−27.741	<0.001
**Northern wheatear**	Elevation	−1.58×10^−4^	7.65×10^−5^	−2.077	<0.038
n = 147	Wing length	0.118	0.007	16.812	<0.001
Age-sex class:	Adult Male	0.000	-	-	-
	Adult Female	0.179	0.049	3.624	<0.001
	Juvenile	−0.87	0.079	−11.018	<0.001
**Black redstart**	Elevation	−0.001	5.34×10^−5^	−18.996	<0.001
n = 215	Wing length	0.103	0.006	17.927	<0.001
Age-sex class:	Adult Male	0.000	-	-	-
	Adult Female	0.596	0.047	12.656	<0.001
	Juvenile	0.494	0.039	12.586	<0.001
**Linnet**	Elevation	−1.68×10^−4^	6.46×10^−5^	−2.616	0.009
n = 199	Wing length	0.068	0.006	10.448	<0.001
Age-sex class:	Adult Male	0.000	-	-	-
	Adult Female	−0.096	0.035	−2.712	0.006
	Juvenile	−0.868	0.033	−26.44	<0.001
**Dunnock**	Elevation	−0.001	2.67×10^−4^	−4.605	<0.001
n = 52	Wing length	0.092	0.012	7.912	<0.001
Age-sex class:	Adult Male	0.000	-	-	-
	Adult Female	0.64	0.071	9.032	<0.001
**Alpine accentor**	Elevation	−0.002	1.91×10^−4^	−10.531	<0.001
n = 71	Tarsus length	0.061	0.007	8.591	<0.001
Age-sex class:	Adult Male	0.000	-	-	-
	Adult Female	0.105	0.062	1.686	0.092
	Juvenile	−1.153	0.049	−23.526	<0.001

See Methods for details.

**Figure 4:**
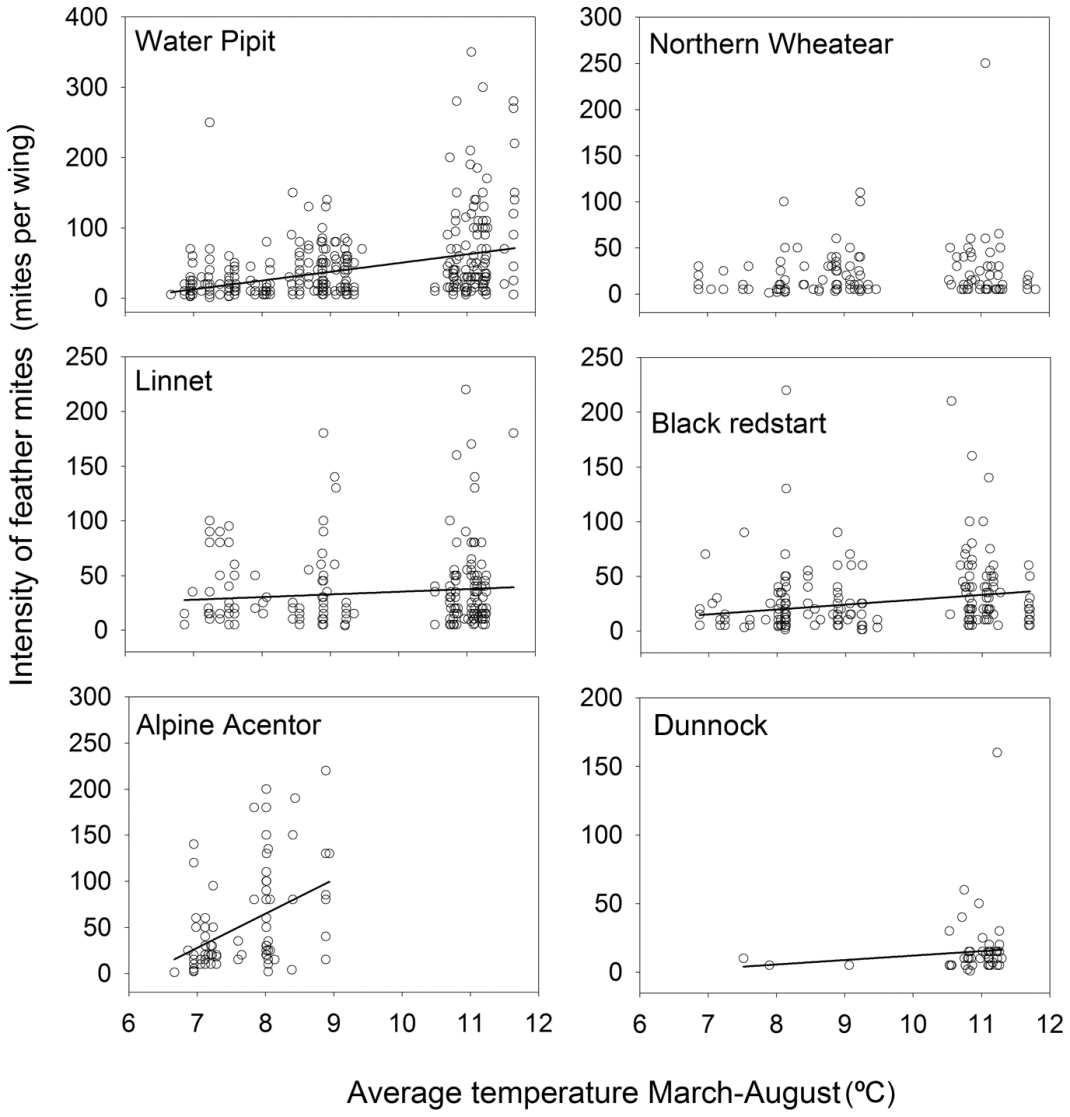
Feather mite intensity and temperature. Relationship between average mite intensity and average temperature (March-August) of the six bird species analyzed. The trend line is shown when the relationship was significant.

Although average temperature and precipitation covaried along the whole elevational range of the northern slope (Fig. 2A), there were no correlation between these climatic variables in our sampling localities (Pearson's r = 0.056, *P* = 0.86, n = 12). Average temperature showed a significant positive relation with mite intensity in five of the six bird species, and was unrelated in the northern wheatear (Table 3, Fig. 4). Accumulated precipitation showed a weaker pattern, being negatively correlated with mite intensity in three out of the six bird species (Table 3). Therefore, the higher mite intensities were found in the warmest sites and, in half of the bird species, also in the driest ones. The percentage of variance explained by climate models for each bird species ranged from 18.2% to 70.2%, and the explanatory power of the average temperature tended to be higher than that of precipitation (Table 4).

**Table 3:** Results of generalized linear mixed models testing for the relationship between mite intensity and climate variables.

		Estimate	SE	*Z*	*P*
**water pipit**	Temperature	0.030	8.63×10^−4^	35.320	<0.001
n = 387	Precipitation	−1.55×10^−4^	1.94×10^−5^	−7.960	<0.001
	Wing length	0.039	0.003	12.450	<0.001
Age-sex class:	Adult Male	0.000	-	-	-
	Adult Female	0.222	0.030	7.310	<0.001
	Juvenile	−0.378	0.024	−15.670	<0.001
**Northern wheatear**	Temperature	0.002	0.002	1.335	0.182
n = 147	Precipitation	4.45×10^−6^	5.10×10^−5^	0.087	0.93
	Wing length	0.191	0.007	16.383	<0.001
Age-sex class:	Adult Male	0.000	-	-	-
	Adult Female	0.170	0.050	3.385	<0.001
	Juvenile	−0.780	0.077	−10.159	<0.001
**Black redstart**	Temperature	0.033	0.001	27.133	<0.001
n = 215	Precipitation	5.73×10^−5^	3.65×10^−5^	1.568	0.117
	Wing length	0.087	0.006	15.534	<0.001
Age-sex class:	Adult Male	0.000	-	-	-
	Adult Female	0.541	0.046	11.712	<0.001
	Juvenile	0.361	0.039	9.166	<0.001
**Linnet**	Temperature	0.005	0.002	3.216	0.001
n = 199	Precipitation	−2.18×10^−4^	4.13×10^−5^	−5.290	<0.001
	Wing length	0.054	0.007	7.662	<0.001
Age-sex class:	Adult Male	0.000	-	-	-
	Adult Female	−0.060	0.037	−1.591	0.112
	Juvenile	−0.739	0.035	−21.41	<0.001
**Dunnock**	Temperature	0.115	0.017	6.689	<0.001
n = 52	Precipitation	−0.001	1.11×10^−4^	−5.873	<0.001
	Wing length	0.050	0.013	3.898	<0.001
Age-sex class:	Adult Male	0.000	-	-	-
	Adult Female	0.934	0.081	11.484	<0.001
**Alpine accentor**	Temperature	0.042	0.004	10.860	<0.001
n = 71	Precipitation	−2.30×10^−5^	4.66×10^−5^	−0.490	0.622
	Tarsus length	0.015	0.023	0.670	0.506
Age-sex class:	Adult Male	0.000	-	-	-
	Adult Female	−0.296	0.045	−6.630	<0.001
	Juvenile	−1.353	0.042	−32.410	<0.001

“Temperature” stands for the average temperature, and “Precipitation” for accumulated rainfall between March and August. See methods for details.

**Table 4:** The importance of each climate variable.

	Model	Percentage of explained variance
**Water pipit**	Only precipitation	18.2
	Only temperature	26.5
	Both	26.7
**Black redstart**	Only temperature	22.5
**Linnet**	Only precipitation	17.1
	Only temperature	17.3
	Both	26
**Dunnock**	Only precipitation	16.4
	Only temperature	17.1
	Both	18.2
**Alpine accentor**	Only temperature	70.2

Percentage of variance explained in the models (see Methods for details) by each climate variable (average temperature and accumulated precipitation between March and August) and by both variables together. Only significant relationships between climate and mite intensity are shown.

**Figure 5:**
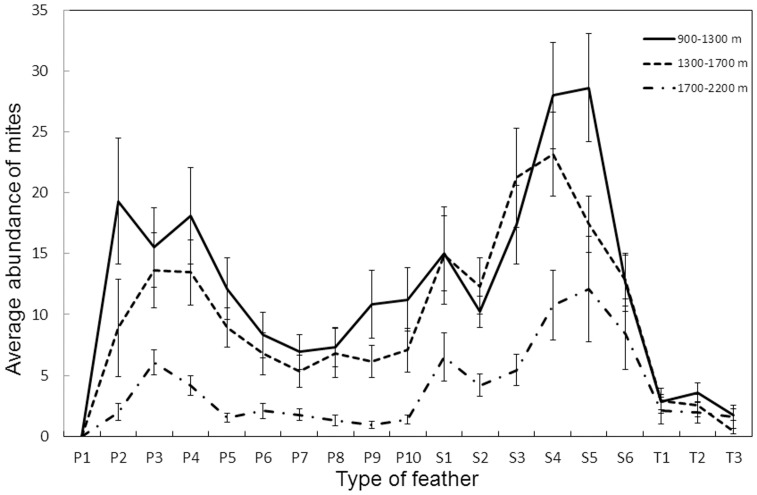
Wing distribution of feather mites along elevation. Average intensity (± SE) of mites per feather (primaries: P1–P10, secondaries: S1–S6 and tertiaries: T1–T3) in three altitudinal bands.

None of the alternative explanations (confounding factors) for mite intensity gradients along elevation in the water pipit could account for the observed patterns. First, only one feather mite species (*Proctophyllodes schwerinensis* [50]) was recorded along the elevation gradient after analyzing 1276 mites from 40 birds from eight localities from 850 to 2050 m.a.s.l. Therefore, a single species lived on the wing feathers of both low and high elevation water pipits, and thus no replacement among mite species occurred. Second, mites were unevenly distributed along the wing of water pipits but this distribution was similar throughout the elevation gradient (Fig. 5), with the majority of mites located on the secondaries (mean number of mites in primary feathers ± SD = 7.64±9.94 mites; secondary feathers: 15.24±13.20; tertiary feathers: 2.28±3.51; Friedman's test: *χ* ^2^ = 7.64, n = 97, *P*<0.001). Thus, no apparent displacement towards inner wing portions at high elevations was observed. Moreover, the percentage of mites on the primaries or tertiaries did not depend on instant air temperature at the moment of bird capture (primaries, r_P_ = −0.137, n = 131, *P* = 0.119; tertiaries, r_S_ = −0.07, n = 131, *P* = 0.426), suggesting that no change in response to external temperature occurred in mite distribution. Finally, water pipit uropygial gland size did not change with elevation (GLMMs: Estimate ± SE  = −0.005±0.003, *t* = 1.580, *P* = 0.119), and was unrelated with feather mite intensity (Estimate ± SE  = −0.653±0.652, *t* = 1.002, *P* = 0.320; see Table S2 for results of complete models). Therefore, at least for the water pipit, the three confounding factors tested did not seem to be implicated in the mite intensity-elevation and -climate correlations found in this study.

## Discussion

We found a consistent decline of mite populations with elevation in the studied mountain bird community. A drop in the average temperature was the factor that better explained this pattern in five of the six bird species examined. Although in our study area average temperature and precipitation covary along the elevation gradient, with lower precipitation and average temperature values on mountaintops, the drop in the average temperature seems to exert higher restrictions on feather mite intensity than precipitation.

Temperature controls every aspect of ectotherm biology and temperatures >15°C, the proposed threshold for mite activity [23], is reached only a few hours a day in the highest study localities (at 2000 m.a.s.l.) in the summer, and the average temperature of these sites is much lower. Hence, mites encounter an unfavorable environment at high elevations, a condition that is exacerbated by the fact that they live on the body surface and in non-blood-irrigated tissues, and are thus exposed to external conditions more than organisms living in contact with irrigated tissues. The effect of temperature may be even more marked on wing mites, which are continuously exposed to air flow during bird movements and long song flight displays. Other studies documented feather mite avoidance of low temperatures [23], [26], a pattern that has also been observed in other ectoparasites [24], [54] and is probably determined by the fact that low temperatures reduce their breeding period length and slow down larvae development [55]. Ultimately, this may have important consequences on the growth rate of populations, and thus on their numbers. The northern wheatear was the only species whose feather mites did not show any significant variation in intensity with the average temperature. While the other community members are resident and undergo short altitudinal displacements below the snowline during winter (authors unpublished data), the northern wheatear is a long distance sub-Saharan migrant that spends five months or less in the breeding areas [56]. Therefore, our results suggest that feather mite populations in this species could be highly influenced by the climate of the wintering areas, and not so much by climate during breeding.

Precipitation showed a negative but weaker impact on mite intensity, and its effect was statistically significant only in three out of six species. These results are in line with the work presented by Dubinin about mite's humidity preferences (see above). Local climate in the study area is characterized by a relatively high precipitation regime even in highlands, and conditions of water stress are rarely experienced by invertebrates living on mountains [57], [58]. Thus, while the average temperature gradient is strong in the study area with elevation, humidity is rather high across the study area, thus potentially precluding to appreciate the negative effect that dry conditions may exert over mites.

Apart from climatic factors, feather mite intensity was also influenced by the age and sex of birds. In five of the six species, yearlings presented lower mite intensity than adults and females higher levels than males. The first result might be explained by the transmission of feather mites from parents to fledglings [59], with intensity increasing progressively during the first months of life [21], while differences between sexes could be due to sexual hormones, which impact the secretion of uropygial oils [28].

In the water pipit, none of the alternative drivers of mite intensity had a significant influence, suggesting that population decline along elevation mirrors a real elevation cline. The distribution of feather mites among wing portions was in fact highly stable along the elevation gradient and did not change with temperature, either the average temperature of the environment or instant air temperature at bird capture. Moreover, no turnover in mite species occurred along the elevation gradient and a unique species (*Proctophyllodes schwerinensis*) was hosted by this bird and its intensity declined with elevation (in spite of the fact that the host inhabits mountains up to the highest elevations [60]). Finally, there was no evidence that the uropygial gland size is conditioning mite intensity, although we cannot discard the possibility that some variation in the composition of lipidic secretions along elevation, or alterations in preening activity as a consequence of cold conditions is occurring. For instance, an increase in the density of preen oils could impede its full spread among feathers, but detailed physiological and behavioral studies would be required to appreciate these effects.

In conclusion, by documenting the strong decline in feather mite intensity along an elevation gradient, we show that the population ecology of ectosymbionts may be constrained by environmental factors that are independent of the host, and that even the warm body of an endotherm may not provide optimal homeothermic living conditions for arthropod symbionts. This finding raises questions on the relationship between mites and typically alpine host species, taking into account that feather mite numbers drop at high elevations even in the water pipit and the alpine accentor, two birds with typical alpine habits that occur at even higher elevations and lower temperatures in other alpine areas of Europe [61]. Detailed studies on the fitness of the two counterparts are necessary to clarify the evolution of this interaction in harsh environments, and to identify the drawbacks of living at high elevations for the symbiont and the functional consequences for the host. Moreover, detailed studies on the phenology of mites are required to test for fluctuations in abundance in response to seasonal changes and their magnitude with respect to spatial variation. Finally, this study provides evidence of the weaker intensity of a specialized interaction in extreme abiotic conditions, a result that is partially in line with the prediction of weaker biotic interactions at the harsh extreme of the stress gradient, and emphasizes that mountain systems are useful laboratories for testing interaction dynamics in heterogeneous environments without the confounding effects of historical legacies within communities.

## Supporting Information

Table S1 **Bird individual condition across elevations of the six species studied.**
(DOCX)

Table S2 **Results of generalized mixed models testing for the relationships between (1) Water Pipit gland size and elevation; (2) Water Pipit feather mite intensity and gland size.**
(DOCX)
