# Supplementary material for: Climate-Driven Variation in the Intensity of a Host-Symbiont Animal Interaction along a Broad Elevation Gradient
Source: PLoS One. 2014 Jul 15;9(7):e101942. doi: 10.1371/journal.pone.0101942 (PMC4099072; doi:10.1371/journal.pone.0101942)
Supplement: Table S1 — Bird individual condition across elevations of the six species studied. (DOCX) [file pone.0101942.s001.docx]

Table S1. Bird individual condition across elevations of the six species studied (as measured by the residuals of bird weight on wing or tarsus length). The massif identity and the age-sex class were entered as random factors. A Gaussian distribution of errors was used.

|  |  | Estimate | SE | *t* | *P* |
| --- | --- | --- | --- | --- | --- |
|  |  |  |  |  |  |
| **Water pipit** | Elevation | > -0.001 | > 0.001 | -0.331 | 0.745 |
| n = 375 | Wing length | 0.305 | 0.086 | 3.512 | 0.005 |
|  |  |  |  |  |  |
| **Northern wheatear** | Elevation | -0.001 | > 0.001 | -2.239 | 0.026 |
| n = 144 | Wing length | 0.259 | 0.058 | 4.475 | 0.001 |
|  |  |  |  |  |  |
| **Black redstart** | Elevation | > -0.001 | 0.001 | -0.606 | 0.545 |
| n = 212 | Wing length | 0.138 | 0.035 | 3.773 | > 0.001 |
|  |  |  |  |  |  |
| **Linnet** | Elevation | > -0.001 | > 0.001 | -2.480 | 0.014 |
| n = 192 | Wing length | 0.195 | 0.032 | 6.026 | > 0.001 |
|  |  |  |  |  |  |
| **Dunnock** | Elevation | > -0.001 | 0.001 | -0.092 | 0.927 |
| n = 51 | Wing length | 0.027 | 0.060 | 0.452 | 0.653 |
|  |  |  |  |  |  |
| **Alpine accentor** | Elevation | 0.004 | 0.002 | 1.780 | 0.080 |
| n = 70 | Tarsus length | 0.290 | 0.089 | 3.257 | 0.002 |
|  |  |  |  |  |  |
