# Supplementary material for: Climate-Driven Variation in the Intensity of a Host-Symbiont Animal Interaction along a Broad Elevation Gradient
Source: PLoS One. 2014 Jul 15;9(7):e101942. doi: 10.1371/journal.pone.0101942 (PMC4099072; doi:10.1371/journal.pone.0101942)
Supplement: Table S2 — Results of generalized mixed models testing for the relationships between (1) Water Pipit gland size and elevation; (2) Water Pipit feather mite intensity and gland size. (DOCX) [file pone.0101942.s002.docx]

Table S2. Results of generalized mixed models testing for the relationships between (1) Water Pipit gland size and elevation; (2) Water Pipit feather mite intensity and gland size. In both models the wing length and the sex-age factor were entered as covariates and the massif identity as a random factor.

|  |  | Estimate | SE | Z | p |
| --- | --- | --- | --- | --- | --- |
|  |  |  |  |  |  |
| **(1) Gland size *vs* elevation** | Elevation | -0.005 | 0.003 | -1.580 | 0.119 |
| n=65 | Wing length | 0.143 | 0.076 | 1.879 | 0.065 |
| Age-sex class: | Adult Male | 0.000 | - | - | - |
|  | Adult female | -4.185 | 6.219 | -0.673 | 0.485 |
|  | Juvenile | 3.082 | 6.567 | 0.469 | 0.699 |
|  |  |  |  |  |  |
| **(2) Feather mite** intensity ***vs* gland size** | Gland size | -0.653 | 0.652 | -1.002 | 0.320 |
| n=65 | Wing length | -0.295 | 0.402 | -0.733 | 0.466 |
| Age-sex class: | Adult Male | 0.000 | - | - | - |
|  | Adult female | -21.915 | 32.129 | -0.682 | 0.498 |
|  | Juvenile | -18.235 | 33.883 | -0.538 | 0.592 |
|  |  |  |  |  |  |
